# Study of the fatigue delamination behaviour of adhesive joints in carbon fibre reinforced epoxy composites, influence of the period of exposure to saline environment

**DOI:** 10.1038/s41598-022-23378-4

**Published:** 2022-11-17

**Authors:** A. Argüelles, I. Viña, P. Vigón, M. Lozano, J. Viña

**Affiliations:** 1 Department of Construction and Manufacturing Engineering, University of Oviedo, Edificio Departamental Oeste N°7, Campus de Viesques, 33203 Gijón, Spain; 2 Department of Materials Science and Metallurgical Engineering, University of Oviedo, Edificio Departamental Este, Campus de Viesques, 33203 Gijón, Spain

**Keywords:** Engineering, Materials science

## Abstract

This work analyses the fatigue delamination and fracture stress behaviour under mode I of adhesive joints made on an epoxy matrix composite material with unidirectional carbon fibre reinforcement and a commercial epoxy-based adhesive. DCB type tests (for mode I) were used with the aim to quantify the influence of the period of exposure to a degradation process in a salt spray chamber, to which the tested samples were subjected, on their fatigue behaviour. For this purpose and after a previous static characterisation of the material in which the critical values of the energy release rate for different exposure periods were determined, the levels of the energy release rate to be applied in the fatigue tests and the exposure periods to be considered (no exposure, exposure during one week and twelve weeks) and a ratio of fatigue stress levels of R = G_min_/G_max_ = 0.1 were defined. From this experimental data, the G-N fatigue initiation curves and the G-da/dN growth curves were obtained. The experimental data obtained, in the fatigue initiation phase of the delamination process, have been treated by means of a probabilistic model based on a Weibull distribution, the application of models of these characteristics has allowed a better interpretation of the experimental results obtained. The most relevant result of the work is that, in general, the fatigue limits obtained for the adhesive joint, under mode I fracture, when subjected to a degradation process in a saline environment, do not translate into a relevant loss of its resistance capacity against this fatigue delamination phenomenon, in its initiation phase. On the other hand, the crack growth rates of the material subjected to different periods of exposure to a saline environment are similar and higher than those obtained for the material without exposure.

## Introduction

The use of high-performance composite materials is a reality in a wide variety of industrial sectors such as the aerospace or automobile industry, in which there is great interest in designing and manufacturing lighter components that allow the reduction of fuel consumption, and consequently, reduce operating costs and greenhouse gas emissions, without interfering with its resilience. Therefore, composite materials are increasingly being used for manufacturing, leaving aside the traditionally used metallic materials, also due to their properties of high mechanical resistance, corrosion resistance, fatigue, impact and thermal stability, but the downside to this type of material is its low delamination resistance. In addition to the composite material itself, alternative joining techniques have become a topic of relevance in the industry, replacing conventional screws and rivets. The study of adhesive joints in fibre-reinforced composites has increased significantly.

When the material is subjected to tests to study the phenomenon of adhesion in relation to the phenomenon of delamination in composite materials, it is necessary to take into account the physical, chemical and mechanical properties^1–6^ of the material to be adhered and in particular, an adequate treatment of the surfaces that will serve as a connecting surface, which can lead to a significant improvement to the adhesion, modifying the initial properties of said surfaces^7–10^, especially in fibre-reinforced polymers due to the low surface tension and wettability they present. Furthermore, it must be taken into account that these types of joints cause an increase in the plastic dissipation energy at the fracture of the adhesive joint^11,12^.

Another important parameter associated to the behaviour of the adhesive joints which is being studied on different materials, including composite materials, is the influence of the type and speed of application of the applied load^13–17^.

In addition, there are lines of work associated to the behaviour of adhesive joints against initiation and growth processes of delamination, in which the properties of the joint, the thickness of the adhesive^18^ and concerning different types of adhesives used are studied^19,20^. Approached with different test methodologies: through pure shear tests^7,21^, or through fracture mechanics, under stress in mode II^22^ and mixed mode (combination between mode I and mode II)^23–25^.

On the other hand, the fatigue behaviour of adhesive joints in mixed materials^26,27^ with the incorporation of reinforced nanofibers^28^: silica nanoparticles^29^ and rubber microparticles^30^ are being studied under different experimental methodologies, also under different fracture modes^31–33^. A major experimental effort is being made to modify the properties of the adhesive bond, by means of laser surface pre-treatments^34^, the incorporation of additional elements to the bond that collaborate in improving the adhesion and they act as a reinforcement of the union against delamination phenomena, the incorporated particles range from carbon nanotubes (CNT)^35,36^ to graphene nanoplatelets (GNP)^37^.

Another line of work in this field analyzes under different fibre orientations of the composite material^38^, also the behaviour of adhesive joints in composite materials subjected to different degradation processes such as humidity and its effects on the delamination process under pure fracture modes^39^, exposure to a saline environment^40,41^, freezing and thawing^42^, water absorption in joints with hybrid composite materials^43^, the effects of temperature^44–46^ and the combination of the effects of humidity and temperature^44,47,48^.

It is worth noting the important contribution that has been made and is being made to provide a tool for the calculation and verification of structures subjected to different loading conditions and manufactured with technologies associated to composite materials joined by adhesives, the knowledge necessary for the numerical simulation of their behaviour and thus be able to develop interesting methodologies and try to provide a numerical solution to this type of joint^49–53^.

The aim of this work is to evaluate the behaviour of adhesive joints against the fatigue delamination phenomenon when they are subjected to different periods of exposure to a saline environment, for which an epoxy matrix composite material with unidirectional carbon fibre reinforcement and an epoxy-based adhesive was selected as a substrate. For the characterisation of the strength of the joint against delamination, the ERR reached by the joint under mode I fracture stress has been taken as a study parameter. Analysing the influence that the applied degradation process, saline environment, has on the adhesive bond in relation to the periods of exposure to which it has been subjected and the influence on its fatigue life, both in its initiation and fatigue crack growth phases.

## Materials used

The materials used are described below, that is, the type and basic characteristics of the used composite material and the type of adhesive used in this study.

### Type of composite material used

The composite material selected in this work as a substrate, is composed of an epoxy matrix and unidirectional carbon fibre reinforcement with the trade name MTC510-UD300-HS-33% RW. Table 1 shows the mechanical properties of the laminate.

**Table 1:** Mechanical properties of the substrate used.

	Elastic modulus^a^		Tensile stress^a^		Shear modulus^b^	Shear stress^b^
Material	E_11_ (GPa)	E_22_ (GPa)	σ_11_ (MPa)	σ_22_ (MPa)	G_12_ (GPa)	τ_max_ (MPa)
MTC510- UD300-HS	122 CV = 8,5%	8,5 CV = 8%	1156 CV = 12,5%	28 CV = 11,8%	5,2 CV = 9,8%	37 CV = 2%
^a^ ASTM D 3039 M^54^ ^b^ ASTM D 3518 M^55^						

The laminate manufacturing process, the basis of this study, and which will then be bonded by means of the selected adhesive, has been produced by vacuum moulding using a procedure similar to those used in some industrial processes. Using the thermal curing cycle recommended by the manufacturer of the material, the reinforcing fibres, which compose the material, were arranged in a unidirectional orientation at 0º.

### Characteristics of the adhesive used

A commercial epoxy-based adhesive, Loctite® EA 9461 was used to join each of the parts that will make up the final laminate and whose surface had been previously treated, as described below. Once the manufacturer’s recommended adhesive curing cycle had been completed, the resulting laminates were machined using a diamond cutting machine to obtain the specimens used in all the tests, of dimensions: nominal width of 20 mm and length of 150 mm with a crack initiation length of 50 mm from load line. The total thickness of each specimen was 4.3 ± 0.1 mm.

A 12 µm thick Teflon PTFE film is placed between the substrates at one of its ends and will act as the initiator of the delamination process.

## Experimental methodology

The most relevant aspects of the experimental programme carried out for the characterisation of the adhesive joint of the selected composite material against the delamination process under mode I fracture stress, both static and dynamic fatigue, when subjected to different periods of exposure to a saline environment are described below.

### Surface preparation

The composite material used as a substrate was surface conditioned by manual abrasive sanding with Al_2_O_3_ sandpaper and grain P220. Once the surface of the composite was treated, it was cleaned and degreased with acetone, for the subsequent gluing process.

### Environmental degradation processes

The purpose of the degradation process, called saline environment in this work, was to evaluate the quality of the adhesive joint subjected to the exposure time to different external agents generated by the process (humidity, temperature and salt concentration).

To do this, a simulated environment has been used in a chamber that speeds up the action of these external agents since they can affect both the adhesive and the compound, favouring a failure in its cohesion, and can affect both the adhesive-substrate interface and its individual components.

Tests have measured an adhesive diffusivity of 6,54,۰10^−7^ mm^2^/s and a saturation time t_sat_/h of 1,59۰10^7^ s/mm or 184 days/mm. In contrast, the values for the composite are 3,5۰10^−7^ mm^2^/s and 6,36۰10^7^ s/mm or 736 days/mm, respectively. This means that the penetration of moisture will be much faster through the side of the specimen occupied by the adhesive than through the composite. However, with the dimensions and exposure time that we are dealing with, it can be assured that saturation has not been achieved except in a very small area of the adhesive.

Knowing the effect that external factors have on the joint make it possible to predict its behaviour in service, as well as to help in the appropriate selection of the materials of which it is composed and to provide solutions to possible problems facing its industrial implementation.

The factors analysed in these tests are exposure period, temperature, humidity and the presence of a saline environment. The influence of these agents is closely related to phenomena such as diffusion, although their effect on the mechanical properties of the adhesive joint also depend on the nature of the adhesive itself and that of the composite material that forms the substrate.

#### Ageing process in salt spray chamber

For the accelerated simulation of the ageing process in a saline environment, a Köheler salt spray chamber, model DCTC 1200 P, was used. The environmental conditions considered were: an average internal temperature of 35ºC ± 2 °C, relative humidity of 89%, an air pressure of 1.2 bars and a saline solution prepared by dissolving sodium chloride quality "p.a.” (reactive for analysis), in demineralised distilled water with a concentration of 50 g/l, a relative density between 1.0255 and 1.04, a pH between 6.5 and 7.2 and a flow rate between 1 and 2 ml/h. At the end of the process, the samples were removed by removing the residual saline solution. The selected periods of exposure in the salt spray chamber were: 1, 2, 4, 12 and 24 weeks.

#### Characterization of the behaviour of the material against delamination

In order to study the influence that the selected ageing process has on the delamination phenomenon, under static and fatigue conditions, of the adhesive joint studied, the energy release rate under mode I fracture stress has been used as a study parameter, using DCB type specimens and carrying out the tests following the test methodology proposed by the ASTM D5528-13 standard^56^, using piano hinges as elements for the application of load to the specimen.

**Figure 1:**
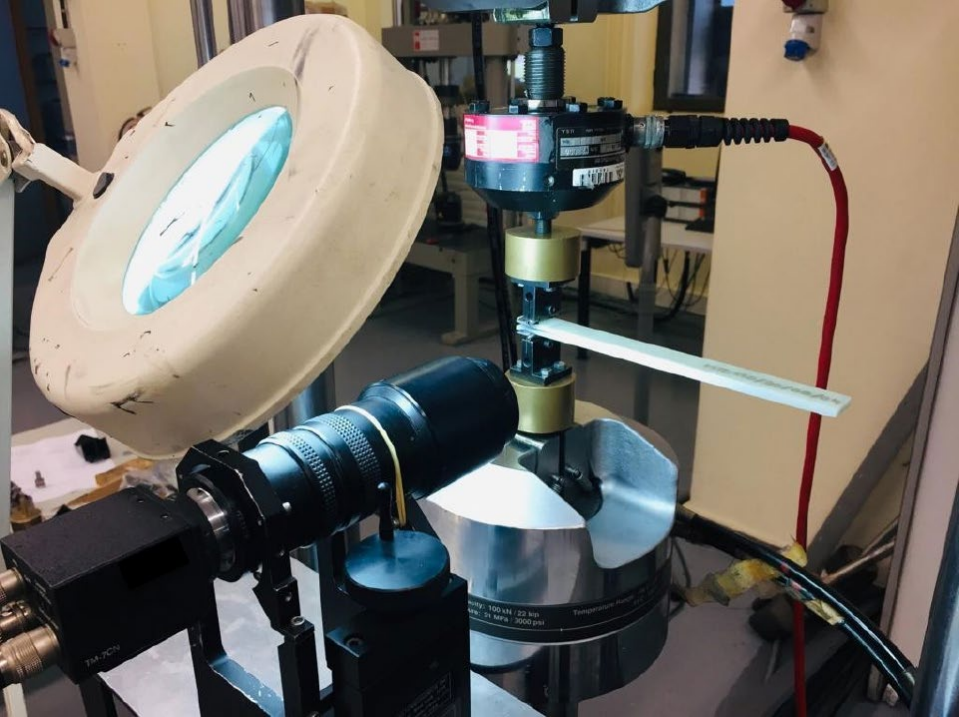
Arrangement of specimen on the test rig.

From the different formulations proposed by this standard, for the determination of the energy release rate under mode I of G_IC_ fracture stress, the modified beam theory (MBT) has been used as the reference formulation for the analysis of its fatigue behaviour, which allows the energy release rate under mode I to be obtained using the following expression:

$$ {\text{G}}_{{{\text{IC}}}} = {\text{3P}}\delta /\left( {{\text{2b}}\left( {{\text{a}} + |\Delta |} \right)} \right) $$

where b is the width of the specimen, P is the applied load, δ is the displacement at the point of load application, a is the crack delamination length and ∆ is a correction factor obtained as a function of flexibility and crack length. The justification for this decision is based on the small difference in values obtained between the three basic formulations proposed by the standard. Figure 1 shows the test configuration.

All specimens were tested using a servo-hydraulic testing machine (MTS Mod. 810) equipped with a 5 kN load cell. Crack advancement was monitored using a high resolution camera.

#### Fatigue characterization

The objective of the experimental fatigue programme was to determine the fatigue behaviour of the adhesive joint tested when subjected to a delamination process under mode I fracture and dynamic stress, with the aim of quantifying the possible influence that the exposure periods in a salt spray chamber have on its behaviour against this phenomenon. Analysing both the initiation phase of fatigue delamination and the subsequent growth phase. Regarding the initiation phase, in this work it has been considered that fatigue failure has occurred in the element when the propagation of an interlaminar crack starts in it and two million cycles have been considered as the fatigue limit for testing.

For the characterisation of the initiation process. The execution of these tests were carried out following the ASTM D 6115–97 standard^57^ at constant stress levels, in function to the values obtained from the previous static characterisation of the material for each of the exposure periods to which they have been subjected, combined with isolated tests. For its definition, the results obtained from the previous static characterisation of the material have been taken as a reference, calculating these levels as percentages of the critical ERR, G_IC_. All fatigue tests were performed with an asymmetry coefficient of R = G_min_/G_max_ = 0.1 and displacement control in the test equipment.

For the study of the crack growth process, the methodology proposed by Stelzer et al.^58^ has been followed, in which, once the previous static characterisation has been carried out, the load and displacement values necessary for the subsequent adjustment of the control in the testing equipment are defined.

## Experimental results and discussion

The results of the experimental study are presented below.

### Static regime

Table 2 shows the results obtained for the adhesive joint studied, indicating the critical ERR calculated under different formulations against the different periods of exposure in a salt spray chamber to which the samples tested (five per exposure period) have been subjected. In addition to the periods selected for fatigue characterisation (no exposure, one week and twelve weeks), the periods of 2, 4 and 24 weeks are presented in order to justify the choice of the periods considered in the dynamic characterisation: since the difference between one and two weeks is of the order of 11%, it can be considered that the results for one week can also be representative for two weeks. Similarly, the results for 12 weeks can be representative for four and twelve weeks.

**Table 2:** Mode I fracture behaviour in function of exposure time in a salt spray chamber.

Periods of exposure	G_IC_ [J/m^2^] (MBT)	G_IC_ [J/m^2^] (CC)	G_IC_ [J/m^2^] (MCC)
No exposure	641 CV = 4,8%	704 CV = 8,3%	642 CV = 6,5%
1 week*	710 CV = 7,6%	921 CV = 9,5%	845 CV = 3,3%
2 weeks	629 CV = 5,6%	664 CV = 11,3%	613 CV = 4,4%
4 weeks	579 CV = 9,1%	612 CV = 7,5%	603 CV = 6,4%
12 weeks	575 CV = 6,3%	595 CV = 4,5%	591 CV = 11%
24 weeks	524 CV = 10,3%	563 CV = 7,4%	532 CV = 7,2%

*MBT* modified beam theory; *CC* compliance calibration method; *MCC* modified compliance calibration method.

*Explanation for these values can be found in reference^40^.

Based on the results obtained, presented in Table 2, the same trend is observed for all the ageing considered, regardless of the formulation used to calculate it, so it is considered reasonable to use as reference values for subsequent fatigue characterisation those obtained using the MBT formulation. It is also observed that the most critical period of exposure corresponds to the one-week period in which higher ERR are reached, in the order of 10% higher than those achieved by the material without any type of exposure, while for exposure periods of 12 weeks there is a drop in the energy release rate in the order of 10.3%. Therefore, the analysis periods considered most representative were: no exposure, 1 and 12 weeks exposure in a salt spray chamber.

### Dynamic regime fatigue

#### Initiation of the fatigue delamination process

In order to improve the reliability in the evaluation of the results obtained in the experimental programme, it was considered convenient to carry out a probabilistic analysis of the whole fatigue life field, for which there are different models^59–61^. In this work, a Weibull regression model proposed by Castillo et al.^62,63^ was used as a statistical tool, which allows the normalisation of the entire fatigue life field and which has already proved to be effective in other cases involving composite materials^64,65^.

**Figure 2:**
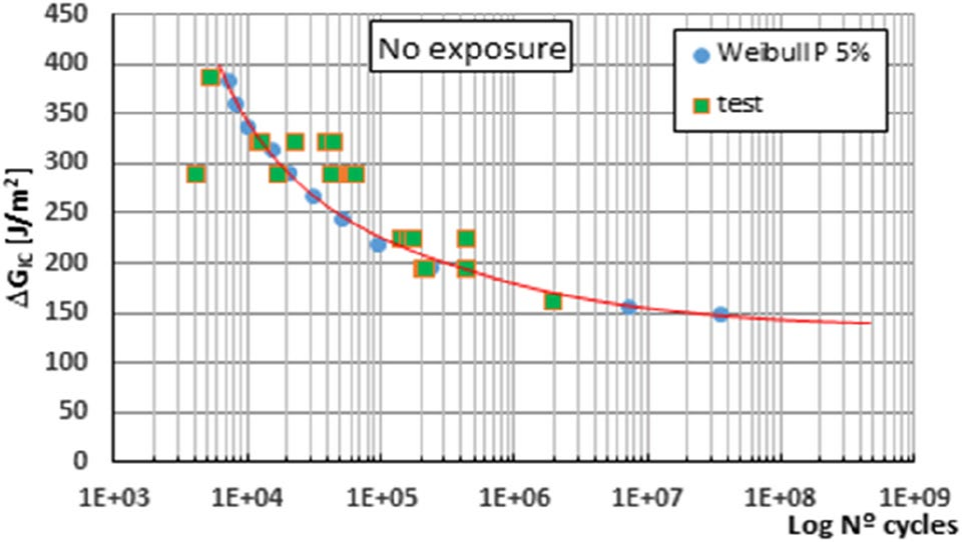
Fatigue initiation curves of material under mode I fracture, for 5% probability of fracture, unexposed material.

**Figure 3:**
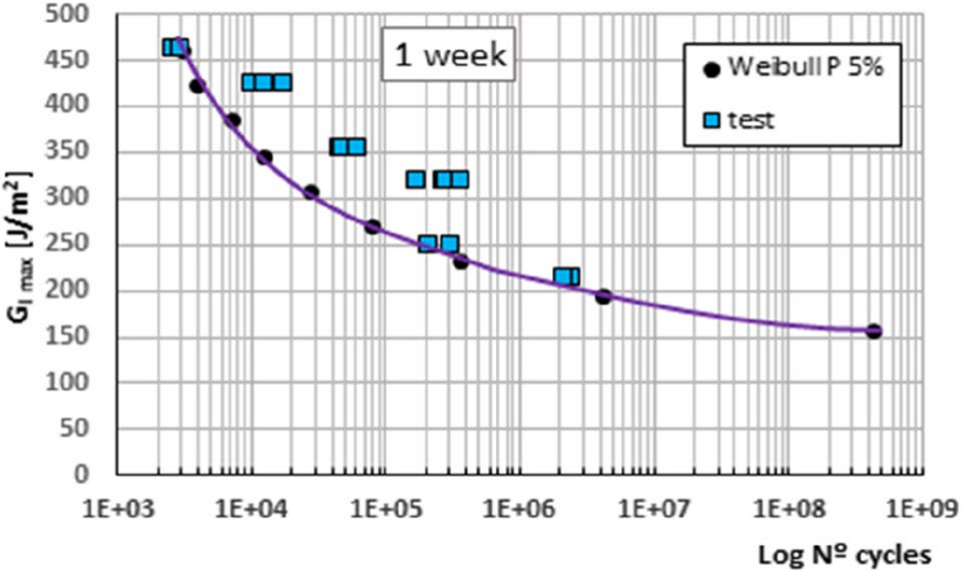
Fatigue initiation curves of material under mode I fracture, for 5% probability of fracture, material under 1 week of exposure.

**Figure 4:**
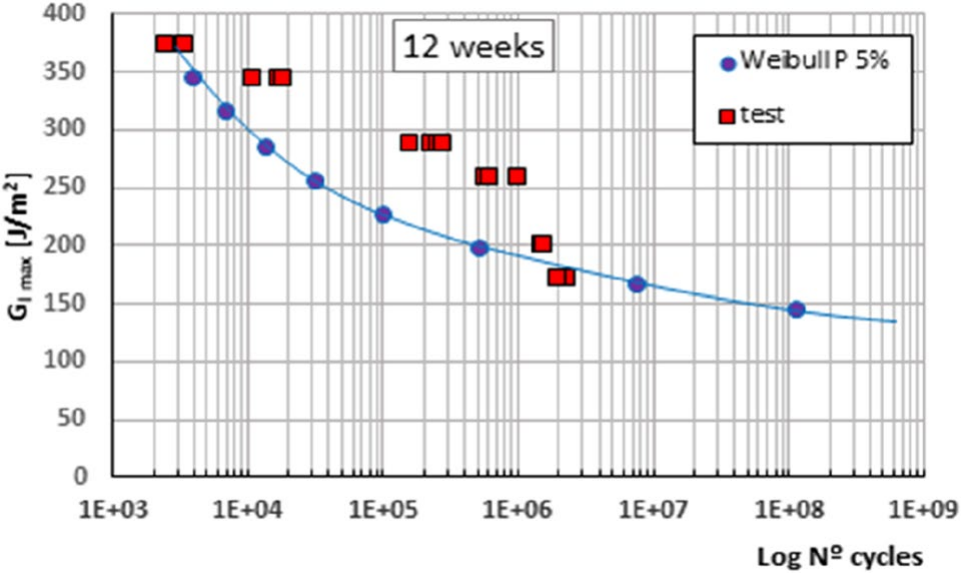
Fatigue initiation curves of material under mode I fracture, for 5% probability of fracture, material under 12 weeks' exposure.

Figures 2, 3 and 4 show the fatigue initiation curves under mode I fracture stress for different periods of exposure of the material in a salt spray chamber: no exposure, one week and twelve weeks respectively and a probability of fatigue failure of 5%, the maximum ERR applied to the specimens tested has been depicted against the number of cycles endured during the fatigue test.

When the maximum ERR applied to the tested samples is considered as a test variable, it can be observed that in the higher number of cycles zone, there is the same tendency for the material in its original state and for the one that has been subjected to a degradation process of 1 and 12 weeks in a salt spray chamber, reaching similar fatigue limits, in the order of 150 J/m^2^. In the area of lower number of cycles there are differences, although not very significant, tending to behaviour similar to those obtained in the previous static characterisation of the material, the same trend for the material without being subjected to any type of exposure and the one subjected to degradation for twelve weeks and somewhat higher values for the case of one week of permanence in a salt spray chamber.

**Figure 5:**
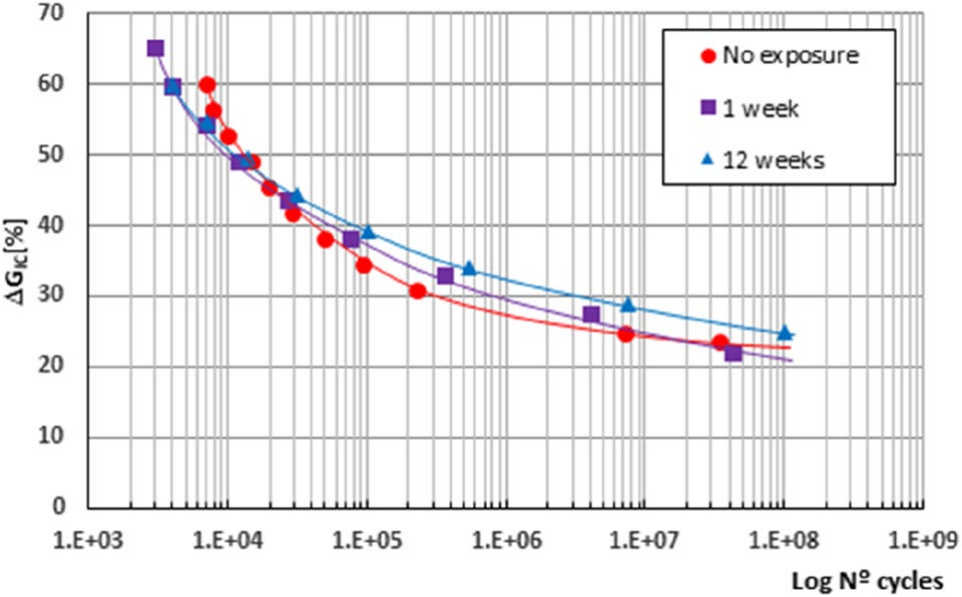
Fatigue behaviour for the three cases studied in function of the stress level.

Figure 5 shows the fatigue curves of the adhesive joints studied for all the processes analysed, showing the stress level expressed in percentage of the critical ERR obtained in the static characterisation of the material versus the number of cycles endured during the fatigue test.

It is observed, in all cases, that their behaviour with respect to the fatigue delamination initiation process does not differ substantially throughout the exposure periods analysed, obtaining similar fatigue curves both for the adhesive joint in its original state and when exposed to a saline environment for different periods of time. Thus, in view of the results obtained and considering the fatigue limit reached as the most representative parameter of their behaviour, when the level of stress applied to the samples tested is considered as a test variable, expressed as a percentage of its ERR reached under static stress, the same tendency can be observed for the material in its original state and that which has been subjected to a degradation process of 1 and 12 weeks in a salt spray chamber, reaching similar fatigue limits, 23% for the original material and 22% for the material subjected to one week in the chamber, slightly higher, 25%, for twelve weeks.

**Figure 6:**
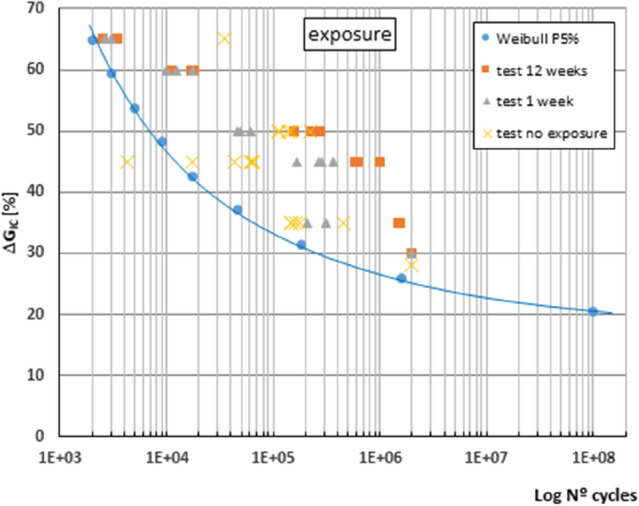
Overall fatigue performance considering all fatigue tests performed on a single specimen.

Figure 6 shows the overall behaviour of the adhesive bond studied considering all the fatigue tests performed as a single representative sample of the behaviour of the material throughout its service life subjected to punctual exposure to a saline environment. The stress level G_IC_ is represented in percentage against its fatigue life.

It is observed that the fatigue limit estimated for infinite life by the statistical model used for the adhesive bond would be in the order of 20% of the ERR obtained from the static characterisation carried out, considering the average of the values obtained in the different exposure periods analysed, it would be 122 J/m^2^, value estimated as the ERR at which infinite life would be reached under fatigue stress.

#### Growth of the fatigue delamination process

**Figure 7:**
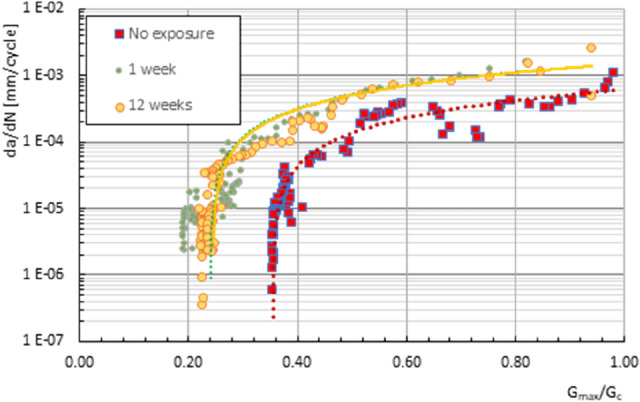
Fatigue crack growth rate versus normalised maximum ERR for the different exposure periods studied.

Figure 7 shows, for some specimens considered representative of the behaviour of the material, the crack growth rate, under fatigue stress, versus the maximum total ERR applied in the dynamic tests with respect to the critical total obtained during the previous static characterisation of the adhesive studied and subjected to the different periods of exposure to the saline environment considered in this work (without exposure, 1 week and 12 weeks). The experimental data obtained and their trend line are presented.

From the experimental data, the same trend can be deduced for the one and twelve weeks exposure periods in the salt spray chamber with similar fatigue delamination growth rates and coincident ERR, in general, higher than the values achieved in the unexposed material for the whole crack growth field.

**Figure 8:**
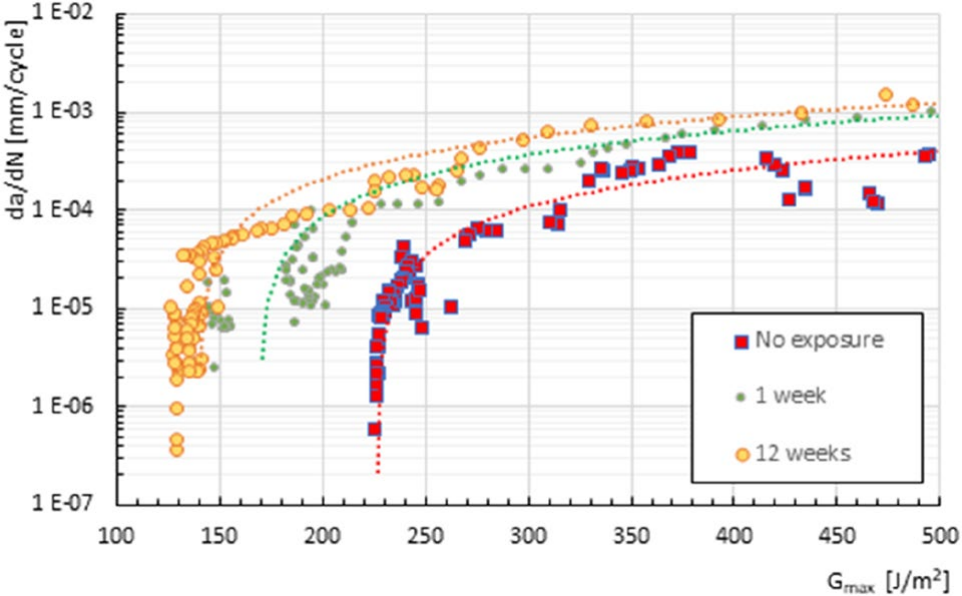
Fatigue crack growth rate versus maximum applied ERR for the different exposure periods studied.

Figure 8 shows the curves representing the crack growth rate against the maximum ERR, calculated using the MBT (modified beam theory) formulation applied to the adhesive joint, in the fatigue crack growth phase, for one and twelve weeks of exposure to a saline environment and without exposure. It is observed that when the maximum ERR applied to the tested specimens is considered as an analysis parameter, the delamination growth is faster as the exposure time to the saline environment increases.

## Conclusions

In this work, some of the parameters controlling the fatigue delamination process under mode I fracture of epoxy-based adhesive bonds in epoxy matrix laminates with unidirectional carbon reinforcement exposed to different periods of time in a saline environment have been investigated experimentally.

- Regarding the initiation of interlaminar cracks in static stress regime and the influence of the exposure time to saline environment, the best behaviour of the adhesive bond for short exposure periods is evident, one week, where ERRs are higher than the values reached by the material without exposure, for longer periods of permanence in the chamber the values reached are lower than those reached by the material without exposure.

- In dynamic mode.

Initiation: The fatigue curves obtained in the initiation phase of the delamination process indicate similar behaviour of the adhesive bond for the two exposure periods studied (1 week and 12 weeks) in which similar fatigue limits are obtained although not very different from those obtained for the material without exposure. When all the tests performed are considered as a single representative sample of the behaviour of the material occasionally exposed to a saline environment, fatigue limits for infinite life of the order of 20% of the ERR obtained under static stress are reached; without considering exposure to a saline environment, the fatigue limit would be 23.5%.

Growth: The rate of interlaminar crack propagation slows down with increasing delamination length, which can be attributed to the presence of fibre bridges and plane changes in the adhesive bond, the delamination zone. The rate of delamination growth increases with increasing applied energy release rate following a trend similar to the Paris law. The exposure time of the adhesive joint studied to a saline environment does not substantially modify the behaviour of the material in its fatigue crack growth phase. The unexposed material performs better in the fatigue crack growth zone than the exposed material, meaning that the crack growth rates are higher when the material is exposed to a saline environment, increasing with exposure time.

## Data Availability

The datasets used and/or analysed during the current study available from the corresponding author on reasonable request.
